# Correlational study on mitochondrial DNA mutations as potential risk factors in breast cancer

**DOI:** 10.18632/oncotarget.8892

**Published:** 2016-04-21

**Authors:** Linhai Li, Lidan Chen, Jun Li, Weiyun Zhang, Yang Liao, Jianyun Chen, Zhaohui Sun

**Affiliations:** ^1^ Department of Laboratory Medicine, Guangzhou General Hospital of Guangzhou Military Command of PLA, Guangzhou, Guangdong 510010, P.R. China; ^2^ Department of Information, No.4 Hospital of PLA, XiNing, Qinghai 810007, P.R. China

**Keywords:** mitochondrial DNA, mutation, high-throughput sequencing, breast cancer

## Abstract

The presented study performed an mtDNA genome-wide association analysis to screen the peripheral blood of breast cancer patients for high-risk germline mutations. Unlike previous studies, which have used breast tissue in analyzing somatic mutations, we looked for germline mutations in our study, since they are better predictors of breast cancer in high-risk groups, facilitate early, non-invasive diagnoses of breast cancer and may provide a broader spectrum of therapeutic options. The data comprised 22 samples of healthy group and 83 samples from breast cancer patients. The sequencing data showed 170 mtDNA mutations in the healthy group and 393 mtDNA mutations in the disease group. Of these, 283 mtDNA mutations (88 in the healthy group and 232 in the disease group) had never been reported in the literature. Moreover, correlation analysis indicated there was a significant difference in 32 mtDNA mutations. According to our relative risk analysis of these 32 mtDNA mutations, 27 of the total had odds ratio values (ORs) of less than 1, meaning that these mutations have a potentially protective role to play in breast cancer. The remaining 5 mtDNA mutations, RNR2-2463 indelA, COX1-6296 C>A, COX1-6298 indelT, ATP6-8860 A>G, and ND5-13327 indelA, whose ORs were 8.050, 4.464, 4.464, 5.254 and 4.853, respectively, were regarded as risk factors of increased breast cancer. The five mutations identified here may serve as novel indicators of breast cancer and may have future therapeutic applications. In addition, the use of peripheral blood samples was procedurally simple and could be applied as a non-invasive diagnostic technique.

## INTRODUCTION

Breast cancer is the most commonly diagnosed malignant tumor among females. Most breast cancers originate in the epithelial cells of lobules and in the non-epithelial tissue of the breast, although occasionally mixed carcinosarcomas can be found [1]. Breast cancers account for more than 1.2 million deaths each year in China alone [2]. Among women in China, breast cancer now has the highest incidence, while in the overall population it is the sixth most deadly cancer [3]. Breast cancer accounts for 12.2% of newly diagnosed cancer cases within China and 9.6% of global cancer related deaths [4]. In China, the mortality rate from cancer is much higher in urban areas than in the rural locales [5], and rising. Thus, there has been considerable effort applied to the development of campaigns for early detection, diagnosis and treatment of breast cancer in urban areas. In addition, investigations into the types and incidence rate of cancer, risk factors and an analysis of the high-risk populations are being supported [6–11].

Breast cancer is a systemic disease and can be widespread in the early stages. While doctors may overlook tumors less than 1 cm in size, studies report circulating breast cancer cells present in blood samples in cases with these small tumors [12]. The majority of metastases are hematogenous metastases, which can generate tumors in all tissues and organs. The aggressive nature of breast cancer metastasis often leads to treatment failure due to cancer spread and recurrence [13]. The ability of breast cancer to metastasize in its early stages, the difficulty of detecting small tumors, and the relatively low survival rates of patients with recurrences make breast cancer a high-priority in the search for early-stage markers.

Recent developments in the search for genetic links to breast cancer have included the discovery of the BRCA genes, many of whose mutations have been mapped [14]. While encouraging, many factors influence the course of the disease and the search for gene mutations that correlate with cancer must continue. The methods used to locate these mutations have included high-frequency mutation screening, exon 11 screening and sequencing monitoring of all exons and adjacent introns [15]. However, a much more comprehensive analysis is required to identify molecular mechanisms in breast cancer and assess high-risk populations further. This would require large sample sizes and a great deal of expensive high-throughput screening.

Using mitochondrial DNA (mtDNA), though, could obtain meaningful results with considerably less resources spent on testing. Human mitochondrial DNA is a double-loop chain of 16.6 kb, with light and heavy chains. MtDNA is the only genetic material outside the nucleus in eukaryotic cells, and is correlated with oxidative phosphorylation. MtDNA contains 13 subunit coding genes [16–18] (Supplementary Table S1) for the oxidative phosphorylation system (OXPHOS), including seven subunits in complex I (ND1, ND2, ND3, ND4, ND4L, ND5, ND6), a subunit in complex III (CytB), three subunits in complex IV (COX1, COX2, COX3) and two subunits in complex V (ATPase6, ATPase8). MtDNA is also capable of encoding 22 ATP tRNAs and 2 rRNAs for protein and ATP synthesis (Supplementary Table S2). MtDNA has no introns. The only non-coding region is the D-loop region (displacement-region), which has been identified as a regulatory region of mtDNA replication and transcription, containing a replication origin and promoter region [19].

Over the past ten years, researchers have found that the mtDNA mutation rate is several times that of nuclear DNA. These mutations include gene deletions, missense mutations, frame-shift mutations and insertions [20]. Both structural and functional features lie behind mtDNA's greater susceptibility to carcinogen-induced mutation. MtDNA mutations can impair normal respiratory function and release large amounts of ROS, increasing the risk of tumorigenesis. Study data suggests that mtDNA has a strong correlation with tumorigenesis [17].

The objective of this study was to investigate the mitochondrial genome association with breast cancer. Many scholars have confirmed that detection of mtDNA mutations in tumor cells is simpler and more reliable than of nDNA, owing to the fact that nuclear genetic damage is often missed in detection schemes, and that the number of copies of mtDNA is 200 times higher than those of the p53 gene [13, 16]. This can reduce sequencing labor and costs significantly. The study screened peripheral blood of breast cancer patients and researched the relationship between germline mutations and breast cancer. It is much more convenient to obtain samples of peripheral blood than breast cancer tissue samples, and it can be directly applied to non-invasive diagnostic techniques. Additionally, various factors of breast cancer occurrence are maternally inherited, possibly via the mitochondria. Using mtDNA markers of inheritable germline mutations for screening in high risk populations will play an important guiding role for the prevention and early diagnosis of breast cancer.

## RESULTS

### Purification and amplification of mitochondrial extracts

After mtDNA was extracted and purified, the Qubit® 2.0 results indicated that the concentrations of mtDNA samples were ≥10 ng/μl, which is in line with the requirements of PCR template concentration. When tested with 1 % agarose gel electrophoresis at 120 V, 25 min, the 16 kb bands were specific and clear, and without degradation or nuclear genomic contamination, which indicates high-purity mtDNA was extracted.

**Figure 1:**
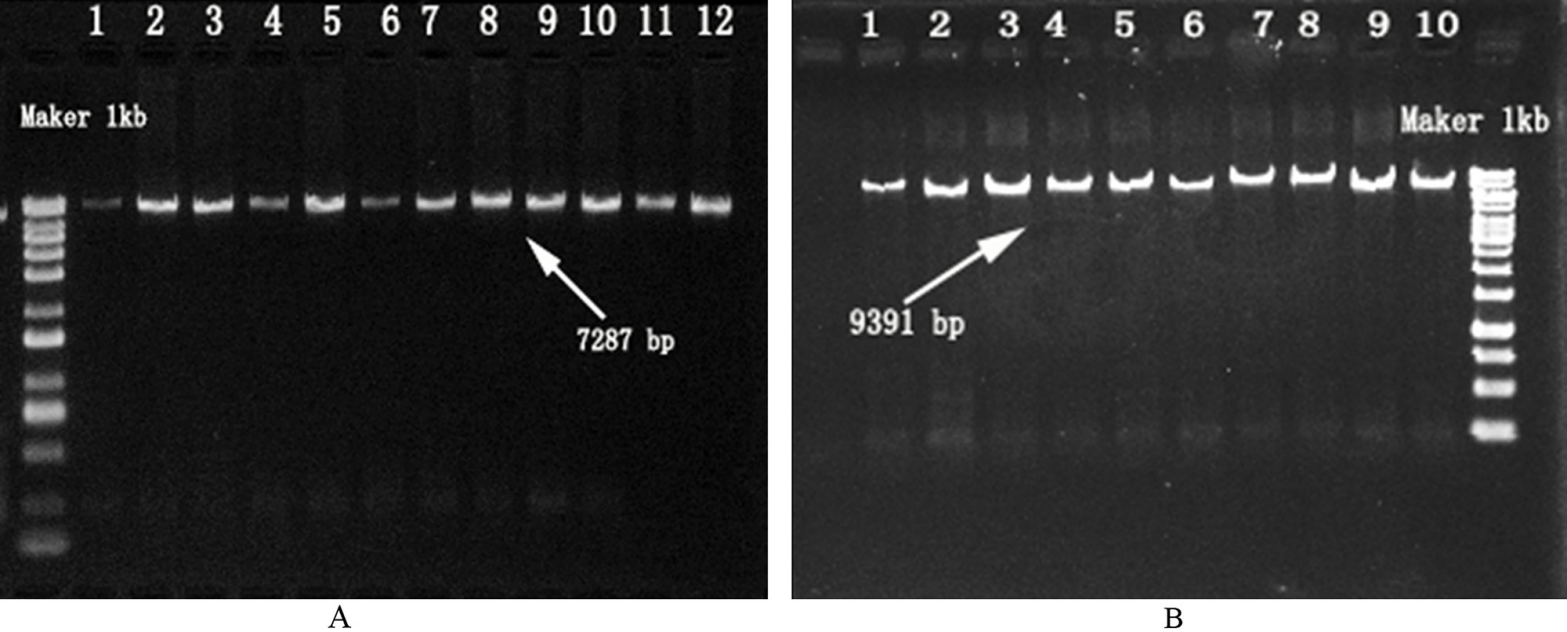
Confirmation of Amplification Products from the Mitochondrial Genome **A.** F7065 / R16455 Primers; Maker: molecular mass marker; Lanes 1-12: samples amplified bands using F7065 / R16455 primers. **B.** F16394 / R7111 Primers; Maker: molecular mass marker; Lanes 1-10: samples amplified bands using F16394 / R7111 primers.

After amplification and gel extraction purification of the mtDNA, their concentrations, as measured by Qubit® 2.0 measurement of the 1-20 coded mtDNA samples indicated a concentration of 50 ng/μl and a volume of 50 ul, which meet the requirements of library construction. The results from the 1 % agarose gel electrophoresis test are shown in Figure 1. As seen in the figure, there are bright bands at 7287 bp (Figure 1A) and 9391 bp (Figure 1B). These bands agreed with expectations, indicating that the acquisition of mtDNA was successful.

### Quality control of the library

**Figure 2:**
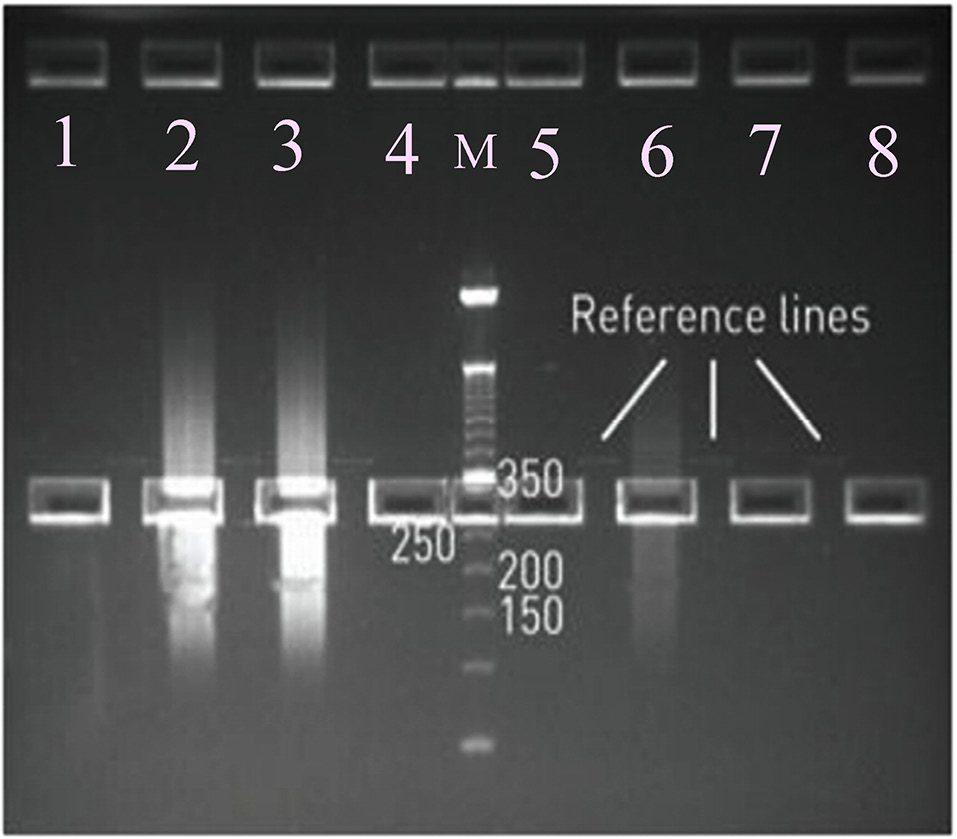
Selections of Mitochondrial Fragment Libraries: The length of sequencing bands: 280-350 bp M: molecular mass marker; Lanes 1, 4, 5, 6, 7, 8: Reference line bands (which were used as the 250-350bp cue lines); Lanes 2, 3: Amplified sample bands.

After the A and P1 ends of mtDNA were connected, the library was formed. Precise quality control of the library is the key to the quality of library sequencing data. The mtDNA library concentrations were measured by Qubit® 2.0 and each library met the requirements (1–10 ng/μl). This sequence had a read length of 200 reactions, interrupted by peak connectors at both ends of 200–250 bp plus 80 bp, which meant the target band was between 280 and 330 bp. Thus we used 2 % low-range detection agarose (120 V, 90 min) and gel extraction was performed to acquire the target band of 280–330bp. Figure 2 shows the correct band.

**Figure 3:**
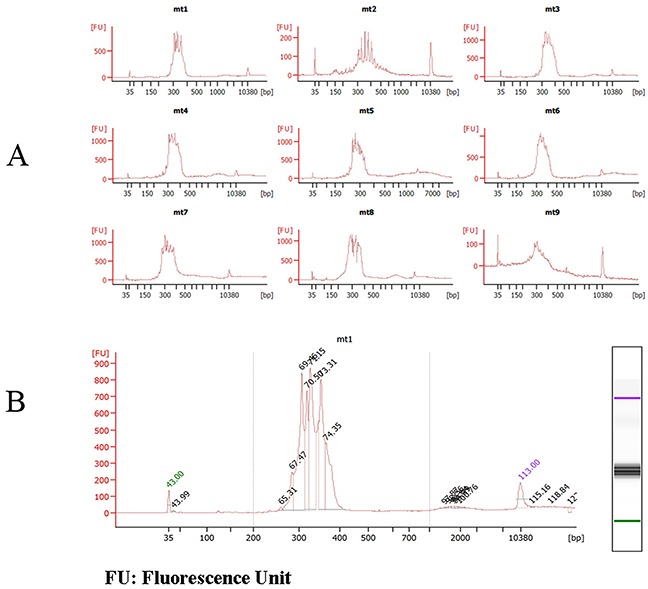
Quality Control Chart of mtDNA Fragment Library Size on Agilent 2100 bioanalyzer **A.** the sizes of some mtDNA fragments **B.** the concentrations of mtDNA1.

The sizes and concentrations of the mitochondrial fragment libraries were confirmed and analyzed by Agilent 2100. The results are shown in Figure 3. Figure 3A indicates that the sizes of the mitochondrial fragment libraries were between 280–330 bp (from mt1 to mt9), which is the expected band range and the same as the agarose gel results.

### Ion Torrent sequencing preliminary results

After using Fast QC-3.4.1.1 software to perform the analysis, the sequencing quality control report was as follows: the average read length of the mitochondrial fragments was 250 bp; and the sequencing quality control between 50 and 250 bp reached the Q20 value. Also, the total base number generated from the original 318 chip sequence data was 1080 Mbp, and the Q20 base number was 920 Mbp. Using 250 bp as the read length of the mtDNA sequence fragments, the coverage of the chip sequencing was 88 %. Stripping out the polyclonal, low-quality data, 61 percent of the data's total read numbers came to 5,096,640. The sequencing quality control between 50 and 250 bp met or exceeded Q20's standards, which indicated that the quality of sequencing data was reliable. This in turn meant the mtDNA sequencing had been successful. The libraries' adapter primer was designed and the library construction kits were successfully assembled, so that the large-scale sequencing model of mtDNA was established.

### Data assembly and comparison

We chose the following software as the analysis tools: Assembler-3.4.2.0, Alignment 2.0, coverage Analysis 3.0, and sequence alignment software based on the Fanse2 algorithm. We assembled and aligned the data using hg38chrM data as the reference genome, and allowed 10 base mismatches. After pre-filtering and aligning the original sequence, we excluded sample data with a filtration rate of less than 40 %, a mapping rate of less than 80%, a depth of coverage less than 100X or where there was uneven coverage. Eventually, 22 cases with valid data from the control group and 83 cases from the disease group were successfully screened. All of these data were used to do the correlation analysis between mutation sites and breast cancer.

### Search of mutation site

In order to perform an SNP loci search to obtain a list of mutations in the samples, we used hg38chrM as our reference genome (NC_012920) and used the Ion Torrent variant caller for MtDNA-3.0 software in combination with the online analysis system (https://ionreporter.LifeTechnologies.com/ir/). Table 1 lists the mutation sites from sample No. 031of the Control Group. The mutation sites for sample no. 137 from the Disease Group are listed in Table 2.

**Table 1:** List of the Mutation Sites in Sample No. 031 of the Control Group

Site	Mutation	Proportion	Site	Mutation	Proportion
46	insA	0.45	7271	A>G	1.00
146	T>C	1.00	8032	C>A	0.37
709	G>A	1.00	8033	delA	0.35
750	A>G	0.96	9123	G>A	1.00
1438	A>G	1.00	9412	G>C	0.29
3109	delT	0.98	9414	delC	0.29
3718	C>A	0.44	9907	G>C	0.29
3721	delA	0.38	9908	delC	0.29
5618	T>C	1.00	10476	C>T	0.26
6305	G>A	0.27	11719	G>A	1.00
6307	delA	0.26	12630	G>T	0.41
6958	G>C	0.68	12631	delT	0.41
6960	delC	0.66	15661	C>T	0.99
7028	C>T	0.99	16518	G>C	0.37
7052	A>G	1.00	16519	T>C	0.39
7078	G>C	0.58	16520	delC	0.27
7079	delC	0.58			

**Table 2:** List of the Mutation Sites in Sample No. 137 of I the Disease Group

Site	Mumtation	Proportion	Site	Mumtation	Proportion
73	A>G	1.00	8032	C>A	0.27
146	T>C	0.99	8033	delA	0.26
199	T>C	1.00	8701	A>G	0.99
263	A>G	1.00	8860	A>G	1.00
489	T>C	1.00	9824	T>C	1.00
523	A>C	0.91	10398	A>G	1.00
524	delC	0.91	10400	C>T	1.00
525	C>G	0.89	11516	C>T	0.28
526	delG	0.88	11517	delT	0.26
750	A>G	0.98	11636	C>A	0.27
1438	A>G	1.00	11637	delA	0.26
2463	delA	0.35	11665	C>T	1.00
3109	delT	0.99	11719	G>A	1.00
3392	G>C	0.36	12091	T>C	1.00
3393	delC	0.36	12630	G>T	0.39
3882	G>A	1.00	12631	delT	0.39
4071	C>T	1.00	12705	C>T	1.00
4611	delA	0.40	13237	delA	0.32
4769	A>G	1.00	15043	G>A	1.00
4850	C>T	1.00	15301	G>A	1.00
5255	C>A	0.39	15326	A>G	1.00
5258	delA	0.39	16092	T>C	0.97
5442	T>C	0.99	16295	C>T	0.98
6455	C>T	1.00	16319	G>A	0.98
6698	delA	0.33	16518	G>C	0.36
6958	G>C	0.51	16519	T>C	0.88
6960	delC	0.49	16520	delC	0.36
7028	C>T	1.00	16528	C>T	0.27
7079	C>T	0.28	16529	delT	0.27

We then calculated statistics for each mutation site, using the number of samples for each locus mutation. The mutation frequency was calculated according to the following formula: mutation frequency = the number of sites with mutation/total number of cases. The results for each site are shown in Supplementary Table S3. These data include 170 mutations detected from the 22 samples in the control group, and 393 mutations from the 83 samples in the disease group. The occurrence of SNP of the disease group is much larger than that of the group control. In addition, the main types of mutations shown in Supplementary Table S3 are point mutations and base deletions, as well as some of the insertion mutations in the base fraction. In addition, a large fragment of 8283 – 8292 bp missing also found in two breast cancer patients' samples. A search on the NCBI database revealed 1321 reported mitochondrial DNA mutations, of which 182 were clearly confirmed to be associated with breast cancer. Our comparison and analysis found 283 mutation sites which had never been reported (indicated as sites with “*” in Supplementary Table S3). These included 232 from the disease group and 88 from the control group.

### Analysis of the correlation between mtSNP and breast cancer

The principle of SNP correlation analysis is to use SNP molecular genetic markers in the overall correlation analysis. Genetic variations in the genome-wide data were selected to do the genotyping and the differences between each genetic variation and frequency variation were compared. Afterwards, we performed a statistical analysis to arrive at the correlation between each variation and target trait, and screened the most relevant genetic variation to validate the results. We then finalized its correlation with the target traits based on the verified results.

### Correlation analysis of breast cancer and mtSNP

**Figure 4:**
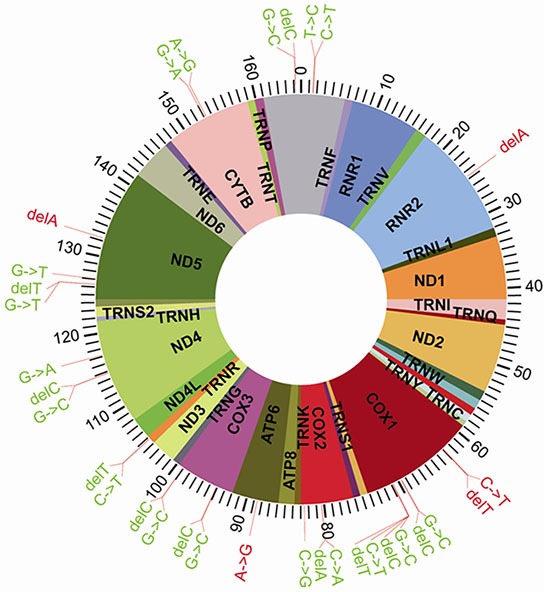
Distribution of the 32 Significantly Different SNPs in the Mitochondrial DNA

SPSS19.0 statistical software was employed to run the χ^2^ test on 2 × 2 tables for each of the corresponding locus mutation frequencies in the two groups. Calculated using Fisher's exact test, we arrived at the test value from the double-sided exact test (p value); if p < α (significance level: α = 0.05), it can be considered that a certain site demonstrates a significant difference between the two groups. Based on this principle, the results shown in Table 3 and Figure 4 indicate that there are 32 mutations with significant differences between these two groups. Previously, 25 of these 32 mutations have not been reported in the literatures.

**Table 3:** Association Analysis between the 32 Significantly Different SNP Mutations and Breast Cancer

Site	SNP	Gene	Mutation frequency		P	OR	95%CI
			Disease group	Control group			
16518*	G>C*	D-loop	0.12	0.36	0.021	0.24	0.080~0.714
16520*	delC*		0.12	0.32	0.046	0.294	0.096~0.895
146	T>C		0.02	0.18	0.017	0.111	0.019~0.654
150	C>T		0.07	0.23	0.05	0.265	0.072~0.970
2463* HR	delA* HR	RNR2	0.28	0.05	0.022	8.05	1.023~63.342
6296*HR	C>T* HR	COX1	0.17	0	0.038	4.464	0.555~35.900
6298* HR	delT* HR		0.17	0	0.038	4.464	0.555~35.900
6958*	G>C*		0.3	0.64	0.006	0.246	0.092~0.661
6960*	delC*		0.3	0.68	0.002	0.201	0.073~0.554
7078*	G>C*		0.08	0.64	0	0.053	0.016~0.168
7079*	delC*		0.08	0.64	0	0.053	0.016~0.168
7079*	C>T*		0.11	0.32	0.039	0.261	0.084~0.809
7081*	delT*		0.02	0.23	0.004	0.084	0.015~0.469
8032*	C>A*	COX2	0.06	0.68	0	0.03	0.008~0.107
8033*	delA*		0.08	0.73	0	0.035	0.010~0.107
8254*	C>G*		0.01	0.14	0.028	0.077	0.008~0.784
8860 HR	A>G HR	ATP6	0.19	0	0.021	5.254	0.658~41.923
9412*	G>C*	COX3	0	0.55	0	0.01	0.001~0.086
9414*	delC*		0	0.55	0	0.01	0.001~0.086
9907*	G>C*		0.06	0.68	0	0.03	0.008~0.107
9908*	delC*		0.06	0.68	0	0.03	0.008~0.107
10476*	C>T*	TRNR	0	0.23	0	0.041	0.004~0.373
10477*	delT*		0	0.09	0.042	0.12	0.010~1.396
11478*	G>C*	ND4	0	0.09	0.042	0.12	0.010~1.396
11479*	delC*		0	0.09	0.042	0.12	0.010~1.396
11719	G>A		0.45	0.73	0.03	0.302	0.107~0.848
12630	G>T	ND5	0.2	0.77	0	0.076	0.024~0.235
12631*	delT*		0.2	0.73	0	0.097	0.033~0.284
12705*	C>T*		0.28	0.64	0.003	0.219	0.081~0.591
13237* HR	delA* HR		0.18	0	0.037	4.853	0.606~38.866
15301	G>A	CYTB	0.13	0.36	0.025	0.267	0.091~0.784
15326	A>G		0.2	0.45	0.027	0.309	0.114~0.835

* means the mtSNP is newly discovered; HR means a high risk factor.

These 32 SNP loci with significant differences were assessed for relative risk, and the OR and 95 % CI values were calculated. The analysis results showed there to be 27 SNPs with OR values of less than 1 among the 32 associated SNPs, and they that they had a negative correlation with the incidence of breast cancer. This indicated that these SNPs are protective factors which can reduce the risk of breast cancer occurrence. Meanwhile, the other five SNPs: 2463 (delA), 6296 (C>T), 6298 (delT), 8860 (A>G), 13237 (delA), whose OR values were 8.050, 4.464, 4.464, 5.254, and 4.853 respectively, were positively related to the development of breast cancer, and were thus considered high-risk factors. The incidence of these SNP may thus increase the chance of breast cancer. These five mutations are all located in the coding region of the mtDNA genome: the deletion in the 2463A site of RNR2, C6296T site mutation and 6298T site deletion in COX1, A8860G site mutation on ATP6, and the 13237A site deletion on ND5. Excepting A8860G (rs2001031), the four others were all discovered for the first time in this study.

### Cell heterogeneity of mtSNP in the two groups

The statistics for the number of reads for each mutation was arrived at by high-throughput sequencing, and the statistical results are shown in Table 3 and Table 4. The cellular heterogeneity of the mutations was assessed in each sample, and the proportion of each mutation in the cells was found to be relatively uniform in most of the samples, without differences between the control and disease groups. Although the mutation rate in some samples was quite different between the two groups, it nonetheless cannot be determined whether or not there is a correlation between these differences and the relative risk of developing breast cancer.

**Table 4:** Heterogeneity of the 32 Significantly Different Mutations

Site	SNP	Mutation frequency	
		Disease Group	Control Group
146	T>C	0.9946	0.997
150	C>T	0.9932	0.9972
2463HR	DelAHR	0.331	0.33
6296HR	C>THR	0.467	-
6296HR	DelTHR	0.4618	-
6958	G>C	0.6194	0.6599
6960	delC	0.6021	0.6468
7078	G>C	0.3763	0.6076
7079	delC	0.3688	0.6045
7079	C>T	0.5943	0.6794
7081	delT	0.3082	0.3052
8032	C>A	0.3156	0.3444
8033	delA	0.3128	0.3225
8254	C>G	0.2781	0.2927
8860HR	A>GHR	0.9999	-
9412	G>C	-	0.3249
9414	delC	-	0.3253
9907	G>C	0.2814	0.2824
9908	delC	0.2819	0.2816
10476	C>T	-	0.2672
10477	delT	-	0.2674
10478	G>C	-	0.2637
10479	delC	-	0.2603
11719	G>A	0.9982	0.9982
12630	G>T	0.4032	0.462
12631	delT	0.402	0.4258
12705	C>T	0.9969	0.9989
13237HR	delAHR	0.315	-
15301	G>A	0.9987	0.9998
15326	A>G	0.9987	0.9991
16518	G>C	0.4551	0.4962
16520	delC	0.4575	0.4804

## DISCUSSION

Typically, there are many DNA mutations in tumor cells. Previous research has often focused on nuclear gene mutations, while mitochondrial gene mutations have attracted considerably less attention. However, more recent studies have found that mitochondria are involved in the occurrence and development of apoptosis and tumorigenesis, which raises many questions on the role of mitochondrial DNA mutations in tumor occurrence and development. Mitochondrial DNA is often in long-term exposure to the active oxygen free radicals generated during oxidative phosphorylation, lacks effective repair mechanisms, and has a high sensitivity to mutation. In addition, mitochondrial genes are without introns and particularly closely spaced. These factors mean that the mutations in any site will affect important functional areas of the mitochondrial genes [21].

Researchers have, in recent years, given more attention to mtDNA mutations and their relationship with breast cancer, in particular because the mitochondria play an important role in the regulation of cell apoptosis. Recently, mtDNA mutations have been found in many solid tumors and hematological malignancies. Polyak et al. (1998) [22] first reported that there were 7 cases with somatic mtDNA mutations in tumor cell homogeneity among 10 cases of colorectal cancer patients. A number of researchers have subsequently reported this kind of mutation in other tumors, including 60% of breast cancers. Many somatic mtDNA mutations have been found in varying degrees, different sites, and different types [23–25]. Tan et al. found that many mtDNA missense mutations of breast cancer have resulted in amino acid substitutions [26], such as T14110C and G14207A gene mutations in ND5 and ND6, respectively. Gallardo et al found that the G6267A mutation, which is involved in encoding COX in the mitochondria, is correlated with breast cancer [27].

In this study, genome-wide association analysis was performed on 22 samples from a control group and 83 samples from breast cancer patients in order to locate germline mutations of mtDNA. We found 32 site mutations associated with breast cancer, including 25 newly discovered mutations. After relative risk analysis based on OR values, 27 of the 32 mutations were found to have a negative correlation probability with breast cancer, and thus may have a protective effect that reduces the risk of breast cancer, while the remaining five mutations were positively related to the development of breast cancer. Compared with the normal group, these five mutations were associated with an increased risk of breast cancer by a factor of between 4 and 9. In addition, these five mutations are all located in the coding region of the mtDNA genome. They are: a deletion in the 2463A site of RNR2, C6296T site mutation and 6298T site deletion at COX1, A8860G site mutation at ATP6, and a 13237A site deletion at ND5. Excepting A8860G (rs2001031), the four others are all previously undiscovered. Mitochondrial DNA codes for important respiratory redox enzymes and RNA. Mutations in this coding region may result in frameshift mutations or missense mutations which lead to the encoded proteins losing their physiological functions; they may also produce a chain reaction, thereby causing disorders of physiological function, and these processes may eventually cause cancer.

The 5 mtDNA mutations identified in this study are likely to affect the synthesis of the respiratory chain. They may also result in increases in nuclear gene mutation and induce disorders of apoptosis by nDNA and mtDNA gene regulation a process which plays a very important role in the occurrence and development of breast cancer [28–30].

The present study differs from previous studies in that it focuses on the relationship between mtDNA mutations and breast cancer. Previous studies often used breast cancer tissues and analyzed the relationship between somatic mutations and cancer. However, the samples collected were often middle or late term, which yields limited guidance on early diagnosis. Additionally, many studies focused on nDNA, requiring the collection and screening of thousands of cases for a genome-wide association analysis. By investigating mtDNA mutations, this study focuses on germline mutation association with breast cancer, reduces the amount of screening required, and identifies biomarkers with potential for use in early detection in high-risk populations.

Due to time constraints, this study collected only 255 samples, of which 22 cases in the control group and 83 cases in disease group were used in the association analysis. While we acquired promising and beneficial results, a larger-scale research project must be performed to confirm the scientific relevance and reliability of our analyses. The five mutations identified in the presented study will be further validated in large-scale future studies.

In conclusion, our study differed from much previous work in investigating the relationship between mtDNA germline mutations and breast cancer. The five mutations we found as a result may be indicators in the prevention of breast cancer, with the added possibility that they may be used in treating breast cancer. Moreover, the use of peripheral blood samples was procedurally much easier to conduct and less invasive, and may be applied to non-invasive diagnostic techniques in the future. Although only 255 samples were collected in our study, our use of high-throughput sequencing of mtDNA had never before been reported in China, and we believe the five key mutations identified in this study will be validated by future large-scale studies.

## MATERIALS AND METHODS

### Samples

Patients (the case group): from January 2013 to June 2014, the department of breast surgery in the General Hospital of Guangzhou Military Command collected 118 peripheral blood samples from patients who had just received their first pathological diagnosis of breast cancer and had never before undergone treatment for the condition. All were female, and between 25 and 84 years old (average: 49.92±11.82 years old). The pathological types included 70 cases of invasive ductal carcinoma, 14 cases of intraductal carcinoma, 16 cases of invasive and intraductal carcinoma, 3 cases of intraductal carcinoma with focal infiltration, 7 cases of mucinous carcinoma, 3 cases of ductal carcinoma in situ, 2 cases of invasive lobular carcinoma, 2 cases of atypical medullary carcinoma, and 1 case of invasive papillary carcinoma. Control group: Samples were collected from 137 individuals at the same hospital and the donors received comprehensive physical examination and imaging to exclude the presence of a variety of tumors. All were women, aged 26-80 years of age (average: 55.17±13.28 years old).

Peripheral blood samples were collected after obtaining the approval of the Ethics Committee of Guangzhou General Hospital of Guangzhou Military Command of PLA and the informed consents of donors. The samples, each 5ml of fresh blood stored with EDTA anticoagulant, were collected from the patients before treatment and from the control group at the same time, and were stored at −70°C.

### Primer design

Two pairs of PCR amplification primers were designed in order to perform a complete enrichment of mitochondrial DNA and in vitro amplification for the extracted total genomic DNA. The lengths of the amplified products were 9391 bps and 7287 bps, both capable of covering the entirety of the amplification products' complete mtDNA sequences. These primer sequences were:

The first site: 7065/16455; the length of amplification product is 9391 bps.

F7065: 5′-GCCATCATAGGAGGCTTCATTCAC-3′;

R16455: 5′-CGGAGCGAGGAGAGTAGCACTC TT-3′

The second site: 16394/7111; the length of amplification product is 7287 bps.

F16394: 5′-CCTTGACCACCATCCTCCGTGAA-3′

R7111: 5′-TAGCCTGAGAATAGGGGAAATCAGT-3′

### Acquisition of mitochondrial DNA

Total DNA was extracted and purified from blood cells using the Gene JET Genomic DNA purification Kit (Thermo, USA), according to the manufacturer's instructions. The concentration of total DNA was detected using the Qubit® dsDNA HS Assay Kit (Invitrogen, USA), according to the manufacturer's protocol. Agarose gel (0.8%) electrophoresis was employed to confirm the purification and detect contamination of the total DNA.

Because the nuclear genome contains many sequences that share some homology with mtDNA, the mitochondrial genome from each sample was first amplified as a series of 14 overlapping 1.5–3 kb PCR products to avoid amplification of the nuclear mitochondrial-like sequences. Each of the reaction mixtures contained 10-50 ng total DNA, 2 U/μl Phusion DNA polymerase (0.5 μl), 5 × Phusion HF Buffer (10 μl), 2.5 mM of each dNTP (8 μl), 10 mM each of the forward and reverse primers (2.5 μl), and ddH_2_O to a final volume of 50 μl. PCR was performed using a thermocycler (4375305, Life Technologies) with the following conditions: 10 s at 95°C, 6 min at 72°C, followed by 30 cycles, and a final extension step of 10 min at 72°C. The resulting products were stored at 4°C. Gel electrophoresis was used to visualize the PCR products and AMPure XP Beads (Beckman Coulter Inc., Brea, CA, USA) were used to remove any contaminants. The Qubit® dsDNA HS Assay Kit (Q32851, Life Technologies, USA) and agarose gel electrophoresis were used to determine the concentration and purity of the 7287 bp and 9391 bp DNA products.

### Construction of mitochondrial DNA library

**Figure 5:**
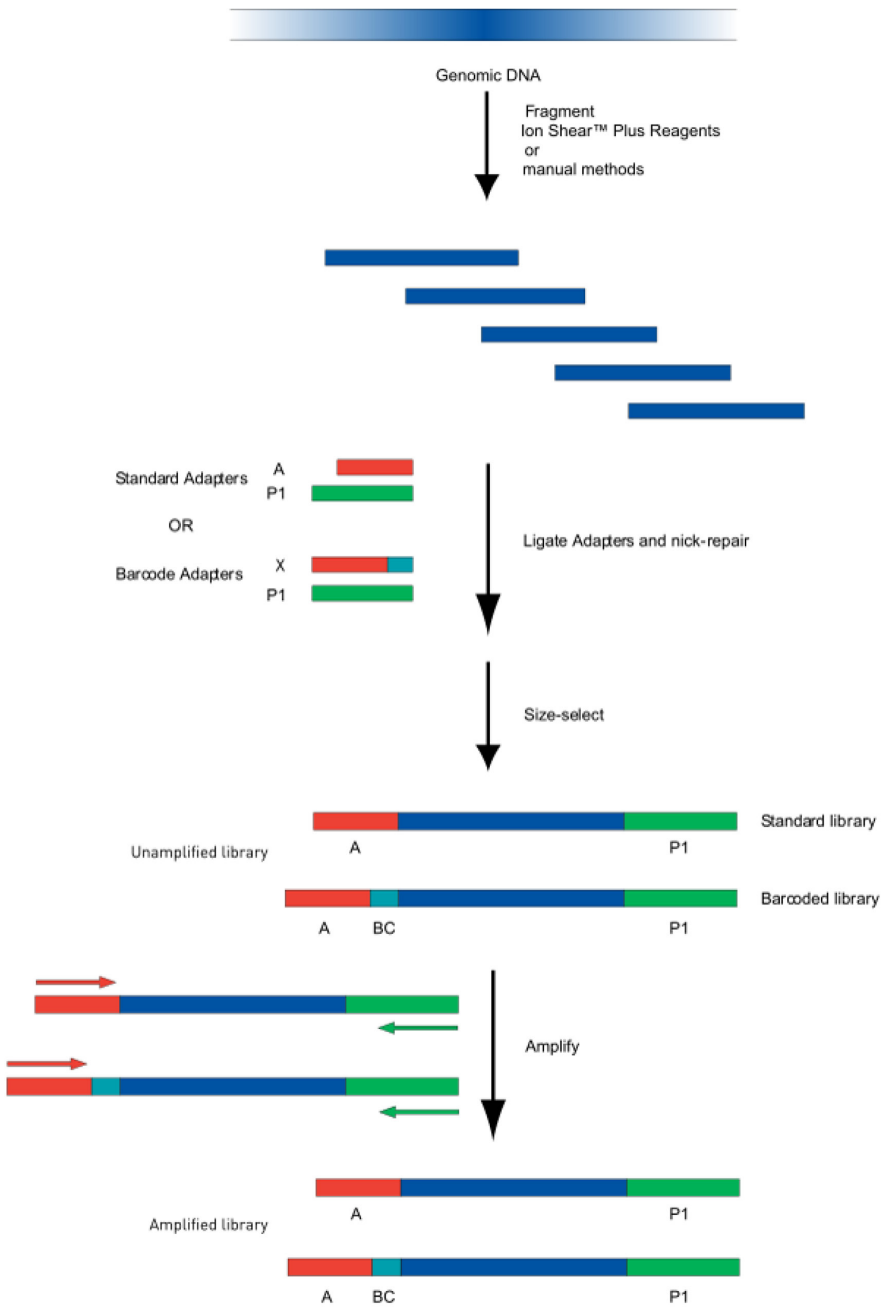
Flow chart of DNA Library Construction Genomic DNA was sheared using a Bioruptor. The sheared fragments underwent end-repair and purified with Agencourt AM Pure reagents. The ends were joined and gaps filled using Ion P1 adapters, Ion Xpress Barcode adapters, DNA ligase and Nick Repair Polymerase. After additional purification, PCR amplification was performed to create amplified libraries.

The 7287 bp and 9391 bp PCR products were mixed equally according to the DNA concentrations in order to create the sample which would be used to construct the mtDNA library. The flow chart of the DNA library construction is shown in Figure 5.

The genomic DNA was sheared using Bioruptor system reagents (Life Technologies, USA). A 2% agarose gel was used to visualize the sample fragment and the main site of cutting. DNA (100 ng in 79 μL) was combined with end repair enzymes (1 μl) in 20 μl 5X end repair buffer and incubated at room temperature for 0.5 hr. Agencourt® AM Pure® reagents were utilized to purify the end-repair DNA. Finally, in order to join the ends and fill the gaps, DNA (25 μl) was mixed with Ion P1 Adapters (2 μl), Ion Xpress Barcode X (2 μl), 10×Ligase Buffer (10 μl), DNA ligase (2 μl), dNTP Mix (2 μl) and Nick Repair Polymerase (8 μl), and nuclease-free water (49 μl), to a total volume of 100 μl. Incubation and thermal cycling were 25 min at 25°C, followed by 5 min at 72°C, then the temperature was held at 4°C. Agencourt® AM Pure® reagents were used, as before, to purify the linked DNA.

The library was amplified via PCR using Platinum PCR Super Mix High Fidelity (100 μl), Library Amplification Primer Mix (5 μl) and 25 μl of the linked DNA. PCR was performed on a thermocycler with the following conditions: 5 min at 95°C, 8-10 cycles at 95°C (15 s), 15 s at 62°C, 1 min at 70°C, an additional 5 min at 79°C and finally hold at 4°C.

After amplification, Agencourt® AM Pure® reagents were used to purify the library. A Qubit dsDNA HS Assay Kit was used to determine DNA concentration and Agilent® High Sensitivity DNA Kit was used to determine the fragment size.

### Template preparation and sequencing

Template preparation was performed with the Ion PGM OneTouch OT2 200 Template Kit (Life Technologies, Carlsbad, CA, USA), according to the manufacturer's instructions. The library was diluted to a final concentration of 26 pM. 20 μL of the 20 code libraries above were mixed with equal volumes of amplification solution and emulsion PCR was performed using the Ion One Touch 2 instrument (Life Technologies, Carlsbad, CA, USA). An Ion OneTouch Enrichment System (Life Technologies, Carlsbad, CA, USA) was utilized to isolate template-positive Ion Sphere Particles, producing an enriched library with 3′ end beads.

The library was sequenced using the Personal Genome Machine (Ion Torrent PGM, Life Technologies, Carlsbad, CA, USA). The results were analyzed with the Ion Torrent PGM server system (Torrent Suite 4.2) which exported raw data along with a sequencing report.

### Correlation analysis and statistical analysis

Based on the Fanse 2 algorithm, we made a comparison using the Homosapiens GRCh38 genome (NC_012920) sequence as the reference and performed mutation site analysis and gene annotation. The mutation frequency was calculated based on the following formula: mutation frequency = number of samples with mutations / total number of samples.

SPSS19.0 (SPSS Inc., Chicago, IL) statistical analysis software was used to analyze the differences between the control group and the patient group, based on the chi-square test, and calculate the mutation frequency.

When the number of samples was not less than 40 (n > 40) and the theoretical frequency was not less than 5 (T > 5), the χ^2^ formula without correction was applied; the Pearson method to calculate the χ^2^is as follows:

$$c^{2}=\sum \frac{{\left(\text{A}-\text{T}\right)}^{2}}{\text{T}}$$

When the number of samples was not less than 40 (n > 40), and the theoretical frequency was between 1 and 5, the χ^2^ calculation was performed with correction. Continuous correction of the chi-square value was calculated using the formula:

$$c^{2}=\sum \frac{{\left(|\text{A}-\text{T}|-0.5\right)}^{2}}{\text{T}}$$

When the number of samples was less than 40 (n < 40), or the theoretical frequency was less than 1, Fisher's exact test was employed. Simultaneously, the relative risk assessment was characterized by the odds ratio (OR) and 95 % confident interval (95 % CI). Using the combined p value of the chi-square test, the correlation between SNP and breast cancer was analyzed so as to screen according to the high risk evaluation index:

When OR = 1, it indicates that the SNP is not associated with breast cancer;

When OR > 1, it indicates that the SNP is a high-risk factor for the occurrence of breast cancer;

When OR < 1, it indicates that the SNP is a protective factor, and could reduce the risk of breast cancer.

## SUPPLEMENTARY TABLES




