# Supplementary material for: Correlational study on mitochondrial DNA mutations as potential risk factors in breast cancer
Source: Oncotarget. 2016 Apr 21;7(21):31270–83. doi: 10.18632/oncotarget.8892 (PMC5058755; doi:10.18632/oncotarget.8892)
Supplement: Supplementary file 1 [file oncotarget-07-31270-s001.pdf]

SUPPLEMENTARY TABLES

Supplementary Table S1: 13 Genes encoding polypeptides

Encoding product	Gene analysis
NADH dehydrogenase(complex I)	MT-ND1
	MT-ND2
	MT-ND3
	MT-ND4
	MT-ND4L
	MT-ND5
	MT-ND6
Coenzyme Q-cytochrome c reductase/cytochrome b (complex III)	MT-Cytb
Cytochrome c oxidase (complex IV)	MT-COX1
	MT-COX2
	MT-COX3
ATP-synthesizing enzyme	MT-ATP6
	MT-ATP8

Supplementary Table S2: Genes encoding 2 Forms of rRNA and 22 Forms of tRNA

Encoding product	Gene analysis
12SrRNA	MT-RNR1
16SrRNA	MT-RNR2
Alanine tRNA	MT-TA
Arginine tRNA	MT-TR
Asparagine tRNA	MT-TN
Aspartate tRNA	MT-TD
Cysteine tRNA	MT-TC
Glutamate tRNA	MT-TE
Glutamine tRNA	MT-TQ
Glycine tRNA	MT-TG
Histidine tRNA	MT-TH
Isoleucine tRNA	MT-TI
Leucine tRNA	MT-TL1<br>MT-TL2
Lysine tRNA	MT-TK
Methionine tRNA	MT-TM
Phenylalanine tRNA	MT-TF
Proline tRNA	MT-TP
Serine tRNA	MT-TS1<br>MT-TS2
Threonine tRNA	MT-TT
Trytophan tRNA	MT-TW
Tyrosine tRNA	MT-TY
Valine tRNA	MT-TV

Supplementary Table S3: Frequency of point mutations in the control and disease groups

See Supplementary File 1
