# Supplementary material for: Correlational study on mitochondrial DNA mutations as potential risk factors in breast cancer
Source: Oncotarget. 2016 Apr 21;7(21):31270–83. doi: 10.18632/oncotarget.8892 (PMC5058755; doi:10.18632/oncotarget.8892)
Supplement: Supplementary file 2 [file oncotarget-07-31270-s002.doc]

**Table S3**  Frequency of point mutations in the control and disease groups

| Site | mtSNP | Control Group | | Disease Group | |
| --- | --- | --- | --- | --- | --- |
|  |  | Case | Mutation frequency | Case | Mutation frequency |
| 27* | C>A* |  |  | 4 | 0.05 |
| 28* | delA* |  |  | 3 | 0.04 |
| 46* | insA* | 1 | 0.05 |  |  |
| 63* | T>C* |  |  | 1 | 0.01 |
| 64* | delC* |  |  | 1 | 0.01 |
| 66* | insT* |  |  | 1 | 0.01 |
| 66* | G>A* |  |  | 1 | 0.01 |
| 73 | A>G | 9 | 0.41 | 32 | 0.39 |
| 93 | A>G |  |  | 1 | 0.01 |
| 94* | G>A* | 1 | 0.05 |  |  |
| 95* | A>T* |  |  | 1 | 0.01 |
| 125 | T>C |  |  | 2 | 0.02 |
| 127 | T>C |  |  | 2 | 0.02 |
| 128* | delC* |  |  | 2 | 0.02 |
| 143 | G>A | 1 | 0.05 |  |  |
| 146 | T>C | 4 | 0.18 | 2 | 0.02 |
| 150 | C>T | 5 | 0.23 | 6 | 0.07 |
| 151* | C>T* |  |  | 2 | 0.02 |
| 152 | T>C | 2 | 0.09 | 10 | 0.12 |
| 183 | A>G | 1 | 0.05 |  |  |
| 185* | delG* | 1 | 0.05 | 2 | 0.02 |
| 189 | A>G |  |  | 2 | 0.02 |
| 194* | C>T* |  |  | 2 | 0.02 |
| 195 | T>C | 2 | 0.09 | 5 | 0.06 |
| 199 | T>C | 2 | 0.09 | 3 | 0.04 |
| 207 | G>A |  |  | 1 | 0.01 |
| 210 | A>G |  |  | 2 | 0.02 |
| 235 | A>G |  |  | 3 | 0.04 |
| 237* | A>G* |  |  | 1 | 0.01 |
| 249* | A>T* | 2 | 0.09 | 8 | 0.1 |
| 250* | delT* | 2 | 0.09 | 8 | 0.1 |
| 263 | A>G | 4 | 0.18 | 24 | 0.29 |
| 309* | delC* |  |  | 2 | 0.02 |
| 316 | insC |  |  | 5 | 0.06 |
| 316 | G>C |  |  | 5 | 0.06 |
| 317 | insG |  |  | 9 | 0.11 |
| 456 | C>T |  |  | 1 | 0.01 |
| 489 | T>C |  |  | 5 | 0.06 |
| 513* | G>A* |  |  | 1 | 0.01 |
| 523* | A>C* |  |  | 11 | 0.13 |
| 524* | delC* |  |  | 11 | 0.13 |
| 525* | C>G* |  |  | 11 | 0.13 |
| 526* | delG* |  |  | 10 | 0.12 |
| 540* | A>C* |  |  | 3 | 0.04 |
| 574* | insC* | 1 | 0.05 | 2 | 0.02 |
| 654* | T>C* |  |  | 1 | 0.01 |
| 663 | A>G |  |  | 3 | 0.04 |
| 681* | T>C* |  |  | 1 | 0.01 |
| 709 | G>A | 6 | 0.27 | 11 | 0.13 |
| 750 | A>G | 18 | 0.82 | 58 | 0.7 |
| 751* | A>T* | 1 | 0.05 |  |  |
| 752 | C>T | 1 | 0.05 | 2 | 0.02 |
| 754* | delA* | 1 | 0.05 |  |  |
| 759* | C>T* |  |  | 1 | 0.01 |
| 827 | A>G |  |  | 2 | 0.02 |
| 979* | C>T* | 1 | 0.05 |  |  |
| 1005 | T>C | 1 | 0.05 | 2 | 0.02 |
| 1009* | C>T* |  |  | 1 | 0.01 |
| 1027* | A>G* | 1 | 0.05 |  |  |
| 1048 | C>T |  |  | 1 | 0.01 |
| 1107 | T>C | 1 | 0.05 | 2 | 0.02 |
| 1119* | T>C* | 1 | 0.05 |  |  |
| 1222* | A>G* | 1 | 0.05 |  |  |
| 1236* | C>T* |  |  | 1 | 0.01 |
| 1310 | C>T |  |  | 1 | 0.01 |
| 1382 | A>C |  |  | 3 | 0.04 |
| 1438 | A>G | 18 | 0.82 | 58 | 0.7 |
| 1520* | T>C* |  |  | 1 | 0.01 |
| 1541 | T>C |  |  | 2 | 0.02 |
| 1736* | A>G* |  |  | 1 | 0.01 |
| 1824* | T>C* |  |  | 3 | 0.04 |
| 1888 | G>A | 1 | 0.05 |  |  |
| 1926* | A>G* |  |  | 1 | 0.01 |
| 1943* | A>G* |  |  | 1 | 0.01 |
| 1978* | A>G* |  |  | 3 | 0.04 |
| 2115* | T>C* |  |  | 1 | 0.01 |
| 2281* | A>G* |  |  | 1 | 0.01 |
| 2308 | A>G |  |  | 1 | 0.01 |
| 2363* | A>G* |  |  | 1 | 0.01 |
| 2399* | A>G* | 1 | 0.05 |  |  |
| 2463* | delA* | 1 | 0.05 | 23 | 0.28 |
| 2464* | delG* |  |  | 1 | 0.01 |
| 2706 | A>G |  |  | 7 | 0.08 |
| 2833 | A>G |  |  | 1 | 0.01 |
| 2835* | C>T* |  |  | 3 | 0.04 |
| 3010 | G>A | 4 | 0.18 | 11 | 0.13 |
| 3109* | delT* | 19 | 0.86 | 61 | 0.73 |
| 3174* | insG* | 1 | 0.05 |  |  |
| 3335* | T>C* |  |  | 1 | 0.01 |
| 3336 | T>C |  |  | 1 | 0.01 |
| 3345* | T>C* |  |  | 7 | 0.08 |
| 3346* | delC* |  |  | 7 | 0.08 |
| 3392* | G>C* | 6 | 0.27 | 18 | 0.22 |
| 3393* | delC* | 6 | 0.27 | 18 | 0.22 |
| 3537* | A>G* |  |  | 1 | 0.01 |
| 3579* | A>G* |  |  | 1 | 0.01 |
| 3591* | G>A* |  |  | 1 | 0.01 |
| 3618* | T>C* | 1 | 0.05 |  |  |
| 3644 | T>C | 0 | 0 | 1 | 0.01 |
| 3666 | G>A | 0 | 0 | 2 | 0.02 |
| 3687* | C>T* | 0 | 0 | 1 | 0.01 |
| 3718* | C>A* | 16 | 0.73 | 39 | 0.47 |
| 3721* | delA* | 16 | 0.73 | 40 | 0.48 |
| 3834 | G>A | 1 | 0.05 |  |  |
| 3882 | G>A | 1 | 0.05 | 2 | 0.02 |
| 3945* | C>T* | 1 | 0.05 |  |  |
| 3970 | C>T | 2 | 0.09 | 13 | 0.16 |
| 4042* | A>G* |  |  | 1 | 0.01 |
| 4048 | G>A |  |  | 3 | 0.04 |
| 4050* | C>T* |  |  | 1 | 0.01 |
| 4071* | C>T* | 2 | 0.09 | 4 | 0.05 |
| 4086* | C>T* | 1 | 0.05 | 5 | 0.06 |
| 4092* | G>A* |  |  | 1 | 0.01 |
| 4129 | A>G |  |  | 1 | 0.01 |
| 4140* | C>T* | 1 | 0.05 |  |  |
| 4164* | A>G* |  |  | 2 | 0.02 |
| 4170* | C>T* |  |  | 2 | 0.02 |
| 4232* | T>C* | 1 | 0.05 |  |  |
| 4248 | T>C |  |  | 1 | 0.01 |
| 4253* | delC* |  |  | 1 | 0.01 |
| 4253* | C>T* |  |  | 6 | 0.07 |
| 4254* | delT* |  |  | 6 | 0.07 |
| 4501* | C>T* |  |  | 1 | 0.01 |
| 4611* | delA* | 3 | 0.14 | 26 | 0.31 |
| 4611* | A>T* |  |  | 11 | 0.13 |
| 4670* | C>T* |  |  | 1 | 0.01 |
| 4703* | T>C* |  |  | 1 | 0.01 |
| 4715 | A>G | 1 | 0.05 | 3 | 0.04 |
| 4768* | T>C* |  |  | 1 | 0.01 |
| 4769 | A>G | 10 | 0.45 | 46 | 0.55 |
| 4811* | A>G* |  |  | 1 | 0.01 |
| 4824* | A>G* |  |  | 3 | 0.04 |
| 4833* | A>G* |  |  | 1 | 0.01 |
| 4850* | C>T* | 2 | 0.09 | 2 | 0.02 |
| 4853* | G>A* |  |  | 1 | 0.01 |
| 4883 | C>T | 3 | 0.14 | 11 | 0.13 |
| 4991* | G>A* |  |  | 1 | 0.01 |
| 5048* | T>C* |  |  | 1 | 0.01 |
| 5054 | G>A |  |  | 1 | 0.01 |
| 5074* | T>C* |  |  | 1 | 0.01 |
| 5093* | T>C* |  |  | 1 | 0.01 |
| 5108* | T>C* |  |  | 1 | 0.01 |
| 5153* | A>G* |  |  | 1 | 0.01 |
| 5178 | C>A |  |  | 9 | 0.11 |
| 5201* | T>C* |  |  | 1 | 0.01 |
| 5231 | G>A |  |  | 1 | 0.01 |
| 5255* | C>A* |  |  | 11 | 0.13 |
| 5258* | delA* |  |  | 11 | 0.13 |
| 5301 | A>G |  |  | 1 | 0.01 |
| 5351* | A>G* |  |  | 1 | 0.01 |
| 5442 | T>C |  |  | 1 | 0.01 |
| 5558 | A>G |  |  | 1 | 0.01 |
| 5580* | T>C* |  |  | 2 | 0.02 |
| 5585* | G>A* |  |  | 1 | 0.01 |
| 5601 | C>T |  |  | 1 | 0.01 |
| 5618* | T>C* | 1 | 0.05 |  |  |
| 5628* | T>C* | 1 | 0.05 |  |  |
| 5752* | A>G* |  |  | 1 | 0.01 |
| 5773 | G>A | 1 | 0.05 |  |  |
| 5783* | G>A* |  |  | 1 | 0.01 |
| 5836* | A>G* |  |  | 2 | 0.02 |
| 5894* | A>G* |  |  | 1 | 0.01 |
| 5900* | A>C* | 1 | 0.05 | 1 | 0.01 |
| 5901* | insA* |  |  | 1 | 0.01 |
| 5913 | G>A |  |  | 1 | 0.01 |
| 5951 | A>G |  |  | 1 | 0.01 |
| 5978* | A>G* |  |  | 1 | 0.01 |
| 6005* | C>T* |  |  | 3 | 0.04 |
| 6007* | T>C* | 1 | 0.05 | 3 | 0.04 |
| 6008* | delC* |  |  | 3 | 0.04 |
| 6009* | delC* |  |  | 3 | 0.04 |
| 6023* | G>A* |  |  | 2 | 0.02 |
| 6179 | G>A |  |  | 3 | 0.04 |
| 6185 | T>C |  |  | 2 | 0.02 |
| 6188* | C>T* |  |  | 1 | 0.01 |
| 6216 | T>C |  |  | 1 | 0.01 |
| 6221 | T>C |  |  | 1 | 0.01 |
| 6296* | C>T* |  |  | 14 | 0.17 |
| 6298* | delT* |  |  | 14 | 0.17 |
| 6302* | A>G* |  |  | 2 | 0.02 |
| 6305* | G>A* | 2 | 0.09 | 1 | 0.01 |
| 6307* | delA* | 1 | 0.05 | 1 | 0.01 |
| 6351* | T>C* |  |  | 1 | 0.01 |
| 6383 | G>A | 1 | 0.05 |  |  |
| 6392 | T>C | 1 | 0.05 | 10 | 0.12 |
| 6413 | T>C |  |  | 1 | 0.01 |
| 6424* | C>T* |  |  | 10 | 0.12 |
| 6425* | delT* |  |  | 9 | 0.11 |
| 6455 | C>T | 2 | 0.09 | 5 | 0.06 |
| 6497* | T>C* |  |  | 1 | 0.01 |
| 6524* | T>C* |  |  | 1 | 0.01 |
| 6575* | A>G* |  |  | 1 | 0.01 |
| 6647* | A>G* |  |  | 2 | 0.02 |
| 6671 | T>C |  |  | 1 | 0.01 |
| 6680 | T>C |  |  | 1 | 0.01 |
| 6698* | delA* | 8 | 0.36 | 31 | 0.37 |
| 6698* | A>G* | 1 | 0.05 | 20 | 0.24 |
| 6699* | delG* |  |  | 1 | 0.01 |
| 6707* | T>C* |  |  | 1 | 0.01 |
| 6752 | A>G | 1 | 0.05 |  |  |
| 6767* | A>G* |  |  | 1 | 0.01 |
| 6788* | A>G* |  |  | 1 | 0.01 |
| 6917* | G>A* | 1 | 0.05 |  |  |
| 6932* | A>G* | 1 | 0.05 |  |  |
| 6958* | G>C* | 14 | 0.64 | 25 | 0.3 |
| 6960* | delC* | 15 | 0.68 | 25 | 0.3 |
| 6960* | C>T* |  |  | 3 | 0.04 |
| 6962 | G>A | 1 | 0.05 | 5 | 0.06 |
| 7028 | C>T | 16 | 0.73 | 42 | 0.51 |
| 7052* | A>G* | 1 | 0.05 |  |  |
| 7078* | G>C* | 14 | 0.64 | 7 | 0.08 |
| 7079* | delC* | 14 | 0.64 | 7 | 0.08 |
| 7079* | C>T* | 7 | 0.32 | 9 | 0.11 |
| 7081* | delT* | 5 | 0.23 | 2 | 0.02 |
| 7196 | C>A | 1 | 0.05 | 1 | 0.01 |
| 7271* | A>G* | 1 | 0.05 |  |  |
| 7346* | A>G* |  |  | 2 | 0.02 |
| 7347* | delG* |  |  | 2 | 0.02 |
| 7598* | G>A* | 1 | 0.05 |  |  |
| 7600* | G>A* | 0 | 0 | 1 | 0.01 |
| 7774* | C>T* | 1 | 0.05 |  |  |
| 7828* | A>G* | 1 | 0.05 | 2 | 0.02 |
| 7861 | T>C |  |  | 1 | 0.01 |
| 7934 | A>G |  |  | 1 | 0.01 |
| 7960* | A>G* | 1 | 0.05 |  |  |
| 8020 | G>A |  |  | 2 | 0.02 |
| 8032* | C>A* | 15 | 0.68 | 5 | 0.06 |
| 8033* | delA* | 16 | 0.73 | 7 | 0.08 |
| 8074* | A>G* | 1 | 0.05 |  |  |
| 8149* | A>G* | 1 | 0.05 | 2 | 0.02 |
| 8200* | T>C* | 1 | 0.05 |  |  |
| 8251 | G>A |  |  | 1 | 0.01 |
| 8254* | C>G* | 3 | 0.14 | 1 | 0.01 |
| 8255* | delG* | 2 | 0.09 | 1 | 0.01 |
| 8281 | C>G |  |  | 2 | 0.02 |
| 8282 | C>A |  |  | 2 | 0.02 |
| 8283-8292 | delCCCTCTAAG |  |  | 2 | 0.02 |
| 8684 | C>T |  |  | 1 | 0.01 |
| 8701 | A>G |  |  | 8 | 0.1 |
| 8794 | C>T |  |  | 1 | 0.01 |
| 8860 | A>G |  |  | 16 | 0.19 |
| 8964* | C>T* |  |  | 1 | 0.01 |
| 9053 | G>A | 1 | 0.05 | 3 | 0.04 |
| 9090* | T>C* | 1 | 0.05 |  |  |
| 9123 | G>A | 1 | 0.05 |  |  |
| 9141* | T>C* |  |  | 1 | 0.01 |
| 9177* | A>G* |  |  | 1 | 0.01 |
| 9180 | A>G | 1 | 0.05 | 2 | 0.02 |
| 9296* | C>T* |  |  | 2 | 0.02 |
| 9377 | A>G |  |  | 1 | 0.01 |
| 9380* | G>A* | 1 | 0.05 |  |  |
| 9412* | G>C* | 12 | 0.55 |  |  |
| 9413* | C>A* | 1 | 0.05 |  |  |
| 9414* | delC* | 12 | 0.55 |  |  |
| 9443* | T>C* |  |  | 4 | 0.05 |
| 9444* | delC* |  |  | 4 | 0.05 |
| 9484* | delT* |  |  | 11 | 0.13 |
| 9484* | T>C* |  |  | 2 | 0.02 |
| 9485* | delC* |  |  | 1 | 0.01 |
| 9490* | C>T* |  |  | 1 | 0.01 |
| 9540 | T>C |  |  | 3 | 0.04 |
| 9548* | G>C* |  |  | 3 | 0.04 |
| 9548* | G>A* |  |  | 1 | 0.01 |
| 9549* | delC* |  |  | 2 | 0.02 |
| 9575 | G>A |  |  | 1 | 0.01 |
| 9612* | G>C* |  |  | 1 | 0.01 |
| 9804 | G>A |  |  | 1 | 0.01 |
| 9824 | T>C | 3 | 0.14 | 3 | 0.04 |
| 9824* | T>A* |  |  | 1 | 0.01 |
| 9845 | T>C |  |  | 1 | 0.01 |
| 9856* | T>C* | 1 | 0.05 |  |  |
| 9907* | G>C* | 15 | 0.68 | 5 | 0.06 |
| 9908* | delC* | 15 | 0.68 | 5 | 0.06 |
| 9950 | T>C |  |  | 2 | 0.02 |
| 9966 | G>A |  |  | 1 | 0.01 |
| 9968* | C>T* |  |  | 1 | 0.01 |
| 10005* | A>G* |  |  | 1 | 0.01 |
| 10031 | T>C |  |  | 2 | 0.02 |
| 10165* | C>T* |  |  | 1 | 0.01 |
| 10167* | delT* |  |  | 1 | 0.01 |
| 10268 | C>T |  |  | 1 | 0.01 |
| 10304 | T>C |  |  | 1 | 0.01 |
| 10310 | G>A | 2 | 0.09 | 6 | 0.07 |
| 10320* | G>A* |  |  | 1 | 0.01 |
| 10397* | A>G* | 1 | 0.05 | 2 | 0.02 |
| 10398 | A>G | 8 | 0.36 | 19 | 0.23 |
| 10399* | delC* |  |  | 1 | 0.01 |
| 10400 | C>T | 8 | 0.36 | 16 | 0.19 |
| 10403* | A>G* | 1 | 0.05 |  |  |
| 10476* | C>T* | 5 | 0.23 |  |  |
| 10477* | delT* | 2 | 0.09 |  |  |
| 10527* | C>T* |  |  | 1 | 0.01 |
| 10535* | T>C* |  |  | 4 | 0.05 |
| 10581* | C>T* |  |  | 1 | 0.01 |
| 10586 | G>A | 1 | 0.05 | 2 | 0.02 |
| 10609 | T>C |  |  | 3 | 0.04 |
| 11038* | delA* |  |  | 3 | 0.04 |
| 11150* | G>C* | 1 | 0.05 |  |  |
| 11151* | delC* | 1 | 0.05 |  |  |
| 11176 | G>A |  |  | 1 | 0.01 |
| 11239* | A>G* | 1 | 0.05 |  |  |
| 11288 | C>T |  |  | 1 | 0.01 |
| 11299 | T>C |  |  | 1 | 0.01 |
| 11353* | T>C* |  |  | 2 | 0.02 |
| 11389* | C>T* | 1 | 0.05 |  |  |
| 11422* | C>T* |  |  | 2 | 0.02 |
| 11478* | G>C* | 2 | 0.09 |  |  |
| 11479* | delC* | 2 | 0.09 |  |  |
| 11516* | C>T* | 4 | 0.18 | 10 | 0.12 |
| 11517* | delT* | 1 | 0.05 | 9 | 0.11 |
| 11524* | A>C* |  |  | 4 | 0.05 |
| 11525* | delC* |  |  | 5 | 0.06 |
| 11539* | C>T* |  |  | 11 | 0.13 |
| 11541* | delT* |  |  | 10 | 0.12 |
| 11636* | C>A* |  |  | 7 | 0.08 |
| 11637* | delA* |  |  | 7 | 0.08 |
| 11665 | C>T | 2 | 0.09 | 1 | 0.01 |
| 11696 | G>A | 1 | 0.05 | 1 | 0.01 |
| 11719 | G>A | 16 | 0.73 | 37 | 0.45 |
| 11722 | T>C | 1 | 0.05 |  |  |
| 11809 | T>C | 1 | 0.05 |  |  |
| 11902* | G>A* |  |  | 1 | 0.01 |
| 11914 | G>A |  |  | 1 | 0.01 |
| 11944 | T>C | 1 | 0.05 |  |  |
| 11963 | G>A |  |  | 2 | 0.02 |
| 12026 | A>G | 1 | 0.05 |  |  |
| 12030* | A>G* |  |  | 2 | 0.02 |
| 12088* | C>A* |  |  | 1 | 0.01 |
| 12091 | T>C | 2 | 0.09 | 1 | 0.01 |
| 12092* | C>A* |  |  | 1 | 0.01 |
| 12191* | C>T* |  |  | 1 | 0.01 |
| 12216* | C>T* |  |  | 1 | 0.01 |
| 12271* | T>A* |  |  | 1 | 0.01 |
| 12274* | delA* |  |  | 1 | 0.01 |
| 12280* | A>G* |  |  | 1 | 0.01 |
| 12285* | T>A* | 1 | 0.05 |  |  |
| 12338 | T>C |  |  | 2 | 0.02 |
| 12425* | delA* |  |  | 5 | 0.06 |
| 12468* | T>C* |  |  | 1 | 0.01 |
| 12561 | G>A |  |  | 1 | 0.01 |
| 12612 | A>G |  |  | 1 | 0.01 |
| 12621* | C>T* |  |  | 1 | 0.01 |
| 12630 | G>T | 17 | 0.77 | 17 | 0.2 |
| 12631* | delT* | 16 | 0.73 | 17 | 0.2 |
| 12634* | A>G* |  |  | 1 | 0.01 |
| 12651* | G>C* | 1 | 0.05 |  |  |
| 12705* | C>T* | 14 | 0.64 | 23 | 0.28 |
| 12732* | T>C* | 1 | 0.05 |  |  |
| 12811 | T>C | 1 | 0.05 | 2 | 0.02 |
| 12817* | C>G* |  |  | 1 | 0.01 |
| 12818* | delG* |  |  | 1 | 0.01 |
| 12850 | A>G |  |  | 2 | 0.02 |
| 12882* | C>T* | 2 | 0.09 | 4 | 0.05 |
| 12892* | T>C* |  |  | 1 | 0.01 |
| 12900* | A>G* |  |  | 1 | 0.01 |
| 12950 | A>G |  |  | 3 | 0.04 |
| 12971* | C>A* | 2 | 0.09 | 12 | 0.14 |
| 12972* | delA* | 1 | 0.05 | 12 | 0.14 |
| 13005* | A>G* | 1 | 0.05 |  |  |
| 13029 | C>T |  |  | 4 | 0.05 |
| 13030* | delT* |  |  | 4 | 0.05 |
| 13145* | G>A* |  |  | 1 | 0.01 |
| 13191* | T>C* |  |  | 1 | 0.01 |
| 13237* | delA* |  |  | 15 | 0.18 |
| 13237* | A>T* |  |  | 6 | 0.07 |
| 13238* | delT* |  |  | 1 | 0.01 |
| 13269 | A>G |  |  | 1 | 0.01 |
| 13287* | C>T* |  |  | 1 | 0.01 |
| 13326* | A>G* |  |  | 1 | 0.01 |
| 13350* | A>G* | 1 | 0.05 |  |  |
| 13368 | G>T |  |  | 1 | 0.01 |
| 13369* | delT* |  |  | 1 | 0.01 |
| 13488* | T>C* |  |  | 1 | 0.01 |
| 13535* | A>G* | 1 | 0.05 |  |  |
| 13563* | A>G* |  |  | 1 | 0.01 |
| 13708 | G>A |  |  | 1 | 0.01 |
| 13759* | G>A* |  |  | 2 | 0.02 |
| 13928 | G>C |  |  | 4 | 0.05 |
| 13958 | G>C |  |  | 2 | 0.02 |
| 13960* | delC* |  |  | 2 | 0.02 |
| 14002* | A>G* |  |  | 1 | 0.01 |
| 14668 | C>T |  |  | 1 | 0.01 |
| 14727* | T>C* |  |  | 1 | 0.01 |
| 14766 | C>T |  |  | 8 | 0.1 |
| 14783 | T>C |  |  | 1 | 0.01 |
| 14920* | C>T* | 1 | 0.05 |  |  |
| 14971* | T>C* |  |  | 1 | 0.01 |
| 14979 | T>C |  |  | 2 | 0.02 |
| 15010* | A>G* |  |  | 2 | 0.02 |
| 15022* | C>T* |  |  | 1 | 0.01 |
| 15040* | C>T* | 1 | 0.05 | 1 | 0.01 |
| 15043 | G>A | 10 | 0.45 | 19 | 0.23 |
| 15067* | T>C* | 1 | 0.05 | 1 | 0.01 |
| 15071 | T>C | 1 | 0.05 | 1 | 0.01 |
| 15172 | G>A | 1 | 0.05 |  |  |
| 15218 | A>G | 1 | 0.05 | 1 | 0.01 |
| 15235 | A>G |  |  | 2 | 0.02 |
| 15236 | A>G | 2 | 0.09 | 2 | 0.02 |
| 15263 | C>T |  |  | 1 | 0.01 |
| 15301 | G>A | 8 | 0.36 | 11 | 0.13 |
| 15314 | G>A |  |  | 1 | 0.01 |
| 15326 | A>G | 10 | 0.45 | 17 | 0.2 |
| 15337 | C>T | 1 | 0.05 |  |  |
| 15346* | G>A* | 1 | 0.05 |  |  |
| 15388* | T>C* |  |  | 1 | 0.01 |
| 15463* | A>G* |  |  | 2 | 0.02 |
| 15487 | A>T |  |  | 1 | 0.01 |
| 15514* | T>C* |  |  | 1 | 0.01 |
| 15529* | C>T* |  |  | 1 | 0.01 |
| 15651* | C>T* |  |  | 2 | 0.02 |
| 15661* | C>T* | 1 | 0.05 |  |  |
| 15724* | A>G* |  |  | 1 | 0.01 |
| 15758 | A>G | 1 | 0.05 |  |  |
| 15784 | T>C | 1 | 0.05 |  |  |
| 15787* | T>C* | 1 | 0.05 |  |  |
| 15862* | T>C* |  |  | 2 | 0.02 |
| 15883* | G>A* |  |  | 2 | 0.02 |
| 15884 | G>A |  |  | 1 | 0.01 |
| 15900* | T>C* |  |  | 1 | 0.01 |
| 15916* | T>C* |  |  | 1 | 0.01 |
| 15924 | A>G | 1 | 0.05 | 1 | 0.01 |
| 16042* | G>A* | 1 | 0.05 |  |  |
| 16092* | T>C* | 1 | 0.05 | 1 | 0.01 |
| 16093 | T>C | 2 | 0.09 | 1 | 0.01 |
| 16108* | C>T* | 1 | 0.05 | 2 | 0.02 |
| 16129 | G>A | 2 | 0.09 | 5 | 0.06 |
| 16140 | T>C |  |  | 1 | 0.01 |
| 16162 | A>G |  |  | 1 | 0.01 |
| 16194* | insC* |  |  | 1 | 0.01 |
| 16223 | C>T |  |  | 4 | 0.05 |
| 16234 | C>T |  |  | 1 | 0.01 |
| 16227* | A>G* |  |  | 1 | 0.01 |
| 16257 | C>A |  |  | 1 | 0.01 |
| 16261 | C>T |  |  | 2 | 0.02 |
| 16266* | C>A* |  |  | 1 | 0.01 |
| 16272* | A>G* |  |  | 1 | 0.01 |
| 16278 | C>T |  |  | 1 | 0.01 |
| 16290 | C>T |  |  | 4 | 0.05 |
| 16292 | C>T |  |  | 2 | 0.02 |
| 16295 | C>T | 1 | 0.05 | 1 | 0.01 |
| 16297* | T>C* |  |  | 1 | 0.01 |
| 16304* | T>C* |  |  | 6 | 0.07 |
| 16311 | T>C |  |  | 3 | 0.04 |
| 16319 | G>A |  |  | 6 | 0.07 |
| 16320 | C>T |  |  | 1 | 0.01 |
| 16335* | A>G* | 1 | 0.05 |  |  |
| 16357 | T>C | 1 | 0.05 |  |  |
| 16359* | T>C* |  |  | 1 | 0.01 |
| 16362 | T>C | 3 | 0.14 | 11 | 0.13 |
| 16381 | T>C |  |  | 2 | 0.02 |
| 16382* | delC* |  |  | 2 | 0.02 |
| 16390 | G>A | 1 | 0.05 | 2 | 0.02 |
| 16394* | C>T* |  |  | 1 | 0.01 |
| 16470* | G>A* |  |  | 2 | 0.02 |
| 16471* | G>A* |  |  | 2 | 0.02 |
| 16473* | G>A* |  |  | 2 | 0.02 |
| 16518* | G>C* | 8 | 0.36 | 10 | 0.12 |
| 16519 | T>C | 8 | 0.36 | 16 | 0.19 |
| 16520* | delC* | 7 | 0.32 | 10 | 0.12 |
| 16527* | C>T* | 1 | 0.05 |  |  |
| 16528* | C>T* | 1 | 0.05 | 6 | 0.07 |
| 16529 | delT | 1 | 0.05 | 6 | 0.07 |

* means the mtSNP newly discovered
